# Value-Added Carp Products: Multi-Class Evaluation of Crisp Grass Carp by Machine Learning-Based Analysis of Blood Indexes

**DOI:** 10.3390/foods9111615

**Published:** 2020-11-06

**Authors:** Bing Fu, Gen Kaneko, Jun Xie, Zhifei Li, Jingjing Tian, Wangbao Gong, Kai Zhang, Yun Xia, Ermeng Yu, Guangjun Wang

**Affiliations:** 1 Pearl River Fisheries Research Institute, Chinese Academy of Fishery Sciences, Guangzhou 510380, China; fub@prfri.ac.cn (B.F.); xj@prfri.ac.cn (J.X.); lzf@prfri.ac.cn (Z.L.); tianjj@prfri.ac.cn (J.T.); gwb@prfri.ac.cn (W.G.); zk@prfri.ac.cn (K.Z.); xy@prfri.ac.cn (Y.X.); gjwang@prfri.ac.cn (G.W.); 2 School of Arts & Sciences, University of Houston-Victoria, Victoria, TX 77901, USA; KanekoG@uhv.edu

**Keywords:** meat quality, muscle hardness, classification model, random forest

## Abstract

Crisp grass carp products from China are becoming more prevalent in the worldwide fish market because muscle hardness is the primary desirable characteristic for consumer satisfaction of fish fillet products. Unfortunately, current instrumental methods to evaluate muscle hardness are expensive, time-consuming, and wasteful. This study sought to develop classification models for differentiating the muscle hardness of crisp grass carp on the basis of blood analysis. Out of the total 264 grass carp samples, 12 outliers from crisp grass carp group were removed based on muscle hardness (<9 N), and the remaining 252 samples were used for the analysis of seven blood indexes including hydrogen peroxide (H_2_O_2_), glucose 6-phosphate dehydrogenase (G6PD), malondialdehyde (MDA), glutathione (GSH/GSSH), red blood cells (RBC), platelet count (PLT), and lymphocytes (LY). Furthermore, six machine learning models were applied to predict the muscle hardness of grass carp based on the training (152) and testing (100) datasets obtained from the blood analysis: random forest (RF), naïve Bayes (NB), gradient boosting decision tree (GBDT), support vector machine (SVM), partial least squares regression (PLSR), and artificial neural network (ANN). The RF model exhibited the best prediction performance with a classification accuracy of 100%, specificity of 93.08%, and sensitivity of 100% for discriminating crisp grass carp muscle hardness, followed by the NB model (93.75% accuracy, 91.83% specificity, and 94% sensitivity), whereas the ANN model had the lowest prediction performance (85.42% accuracy, 81.05% specificity, and 85% sensitivity). These machine learning methods provided objective, cheap, fast, and reliable classification for in vivo crisp grass carp and also prove useful for muscle quality evaluation of other freshwater fish.

## 1. Introduction

Fish are a valuable source of high-quality animal protein throughout the world, with its annual consumption outpacing population growth between 1961 and 2016 [1]. Grass carp (*Ctenopharyngodon idella*), the largest freshwater fish species, has a global production of about six million tons [1]. Crisp grass carp (*Ctenopharyngodon idellus* C. et V) is one of the most representative varieties of grass carp that shows improved textural characteristics (hardness, chewiness, springiness, etc.) after being fed solely with whole faba bean (*Vicia faba* L.) for 90 to 120 days [2,3]. Crisp grass carp has been deemed a value-added product and is protected as a “China Geographical Indication Product”. The fillets of crisp grass carp are exported to various countries in Southeast Asia and Latin America as well as Hong Kong [4].

Hardness is the most prominent quality indicator of crisp grass carp and is directly related to the consumer’s acceptability [3,4]. As mentioned above, faba bean feeding for 90–120 days is used to improve textual characteristics of crisp grass carp. However, according to aquaculture experiences, approximately 5% of the treated fish still exhibit low muscle hardness similar to ordinary grass carp after 120 d of faba bean feeding, which financially affects producers, regulatory agencies, and consumers. To prevent this, it is necessary to assess the muscle hardness of crisp grass carp products over different culture periods. Sensory evaluation has been the primary method for the evaluation of the muscle hardness of crisp grass carp [5], but this method is subjective and is greatly influenced by the experience of the evaluator [6]. Yang et al. [4] proposed an alternative method for evaluating the muscle hardness of crisp grass carp via instrumental texture analysis, but its widespread application is limited because of high equipment costs and long preparation and analysis times. As such, it is necessary to develop objective, cheap, fast, and reliable in vivo analytical methods for analyzing muscle hardness of crisp grass carp.

Machine learning techniques have emerged as a potential in vivo analytical tool. Machine learning identifies patterns in large datasets and aids in predicting outcomes based on various algorithms [7], which have been applied to classify aquatic animals. For example, support vector machine (SVM) can differentiate between organically and conventionally farmed salmon with an accuracy of 98.2% based on hyperspectral imaging and computer vision [8] and can obtain 82% accuracy using skin images [9]. Additionally, multi-class SVM achieved a high accuracy (97.77%) in classifying six freshwater fish species using skin color and texture [10]. The artificial neural network (ANN) achieved 91.86% accuracy in the automated identification of fish species when combined with machine learning algorithms [11]. In living cattle, muscle quality was evaluated by machine learning based on blood analysis, and the random forest (RF) model distinguished organic cattle with a classification accuracy close to 90% [12]. However, machine learning combined with blood analysis has yet to be applied to predict fish quality.

Therefore, to develop objective and reliable in vivo analytical methods for analyzing the muscle hardness of crisp grass carp, taking advantage of machine learning techniques, the present study first evaluated the quality (muscle hardness) of crisp grass carp. Samples selected based on muscle hardness were used for the analysis of seven blood indexes. Six machine learning models were applied to predict muscle hardness of grass carp based on the training and testing datasets obtained from the blood analysis. The performance of the machine learning methods in classifying muscle hardness was evaluated in view of accuracy, sensitivity, specificity, and the area under the receiver operating characteristic curve (AUC). This work will establish objective, cheap, fast, and reliable in vivo analytical methods for evaluating freshwater fish quality.

## 2. Materials and Methods

### 2.1. Experimental Fish and Sample Collection

The feeding trial of grass carp was conducted at the Pearl River Fisheries Research Institute (Guangdong, China). A total of 540 fish (512.12 ± 10.67 g) were randomly distributed into 6 experimental tanks (tank size: 4 × 4 × 1.5 m) comprising a crisp grass carp group and ordinary grass carp groups (per group in triplicate). The crisp grass carp and ordinary grass carp were fed solely with faba bean and a commercial diet, respectively, for 120 days (d). Thirty-three individuals were sampled from both the crisp grass carp group and ordinary grass carp on 30, 60, 90, and 120 days, and total 264 fishes were used. Two-milliliter blood samples were drawn for blood cell analysis from the caudal vein using a sterile heparinized syringe and immediately transferred to tubes containing ethylenediaminetetraacetic acid (EDTA) that prevents blood from clotting. The 2 mL whole blood was stored at 4 °C for 3 h followed by centrifugation at 3500× *g* for 10 min. The separated serum was stored at −80 °C for biochemistry analysis.

For muscle sampling, the fish were firstly anesthetized with tricaine methanesulfonate (MS-222). Each fish was killed, and the scale, skin, and red muscle were removed. For the texture determination and sensory evaluation, the dorsal white muscle (2 × 2 × 1 cm) was sampled at the junction of the dorsal fin and the lateral line scales from the right and left sides of the fish, respectively.

The experimental protocols used in the present study were approved by the Animal Ethics Committee of the Guangdong Provincial Zoological Society, China (permit number GSZ-AW012).

### 2.2. Muscle Hardness Measurement and Sensory Evaluation

As hardness is one of the key texture indicators for crisp grass carp muscle, we mainly measured muscle hardness using a Universal TA texture analyzer (Tengba instrument company, Shanghai, China) in a double compression Texture Profile Analysis (TPA) test. Each sample was treated using a flattened cylindrical probe (3.5 cm diameter) moving at 1 mm·s^−1^ to compress the tissue to 25% of its original height at room temperature. TPA was performed at least three times for each fillet.

Sensory evaluation of crisp grass carp was performed referring to the procedure of Yang et al. [4]. Texture properties were assessed by a panel of 5 trained experts (male, ages 35–50) using a five-class scale rating test (first level—minimal hardness; second level—moderate hardness; third level—normal hardness; fourth level—high hardness; fifth level—maximal hardness). Prior to the sensory evaluation, the muscle samples were cut into small chunks (2 × 2 × 2 cm), steamed over boiling water for 15 min, and cooled down to room temperature. Before evaluating each sample, the panelists rinsed their mouths five times with water to prevent interference from the previous samples. The final results of the sensory evaluation required a minimum of three identical ratings to be included.

### 2.3. Outlier Samples Removal

In general, crisp grass carp is characterized by muscle hardness greater than 1000 g after being fed faba bean for a couple of months. To improve the accuracy of the established models, muscle hardness was analyzed for all samples, with outlier samples removed based on a muscle hardness boxplot. In the boxplot, points below Q1 − αIQR and above Q3 + αIQR are considered as hardness outliers, where IQR is the interval quartile range, Q1 and Q3 indicate the first and third quartiles, respectively, and α is defined as 1.5 [13].

### 2.4. Measurement of Blood Indexes

Red blood cells (RBC), platelet count (PLT), and lymphocyte (LY) from whole blood samples were measured using a hematology analyzer Mek-7222K (Nihon Kohden, Tokyo, Japan) according to the manufacturer’s instructions. The serum samples were used for measuring hydrogen peroxide (H_2_O_2_) (Kit No. A064-1), glucose-6-phosphate dehydrogenase (G6PD) (Kit No. M015), glutathione (GSH/GSSH) (Kit No. A006-1-1), and malondialdehyde (MDA) (Kit No. A003-1-2) using detection kits from Nanjing Jiancheng Bioengineering Institute (Nanjing, China).

### 2.5. Description of the Algorithms

To construct machine learning-based classification models, the datasets from the blood indexes were divided into two datasets—training and testing datasets. The training dataset was formed from 60% of the total samples used for calculating the classifiers, with the remaining 40% used for the testing dataset to validate the constructed models. Data processing was done using the Python3 sklearn package, and the algorithms used in this study were executed under default settings [14].

We first used unsupervised principal component analysis (PCA) to visualize the natural data distribution in a reduced dimensional space, which also allowed us to verify the relationship between the variables in the multidimensional space [15,16]. After PCA, the association patterns of variables can be clearly described [17].

Following the unsupervised PCA, we applied six supervised learning methods to build models that predict the hardness of crisp grass carp muscle from blood parameters. The models include two linear methods, linear Support Vector Machine (SVM) and Naïve Bayes (NB), and four non-linear models, Gradient Boosting Decision Tree (GBDT), Artificial Neural Network (ANN), non-linear SVM, and Random Forest (RF). Supervised algorithms require labeled training data to generate reasonable classifications for new data, whereas unsupervised algorithms do not.

Gradient boosting decision tree (GBDT) is an algorithm that consists of multiple decision trees, in which the final conclusion is derived from all of the decision trees [18]. The base learner of GBDT is the categorical regression tree (CART), which is a binary tree-based machine learning algorithm that can handle both regression and classification problems.

Artificial neural networks (ANNs) are adaptive non-linear decision-making tools inspired by the structure of the human brain [19]. ANNs consist of a number of nodes connected to each other, which mimic neurons in the human brain, which receive signals from the input links. Each input link (corresponding to a synapse) has an assigned weight that corresponds to synaptic efficiency. ANNs are typically trained by back-propagation consisting of at least three layers: input, output, and the hidden layer that connect the two layers.

Support vector machine (SVM) is a supervised model generally used for sample classification and regression [20]. This algorithm conducts non-linear transformation of the data to fit them into a K-dimensional hyperplane (K > original dimension). The SVM shows an excellent generalization ability when a specialized learning procedure is applied [21].

Partial least squares regression (PLSR) is a standard multi-linear regression model. This model is able to find linear relationships between observable variables and predicted variables [22]. This method is particularly useful when the data suffers from the multicollinearity [23] because it can reduce the number of observable variables and extracts a number of components like PCA.

Naïve Bayes (NB) is a simple algorithm that requires a small amount of data for training because it can be trained very efficiently by supervised learning [24]. The theoretical base of this algorithm is the Bayes theorem, in which each variable is treated as an independent variable.

Random forest (RF) can be used for either classification or regression through the construction of many decision trees [25]. The RF method performs a bootstrap sample from the training dataset and makes a decision tree using each of them. The final prediction is made by the set of trees [26].

### 2.6. Classification Performance, Statistical Analysis, and Calculations

In this paper, the results were validated by a tenfold cross-validation procedure. For this purpose, four indicators were calculated: accuracy, sensitivity, specificity, and the area under receiver operating characteristic curve (AUC). We calculated both micro- and macro-averages of the performance metrics as well as the confusion matrices for each model to present their predictive capabilities.

The performance measures are defined as follows. TP, TN, FP, and FN stand for true positives, true negatives, false positives, and false negatives, respectively.

Accuracy refers to the average number of samples properly categorized.

$$\mathrm{Classification}\;\mathrm{accuracy}\;=\frac{\left(\mathrm{TN}\;+\;\mathrm{TP}\right)}{\;\left(\mathrm{FN}\;+\;\mathrm{TP}\;+\;\mathrm{FP}\;+\;\mathrm{TN}\right)}\;\times \;100\%$$

Sensitivity is the ability to correctly classify samples (i.e., the fraction of target samples correctly classified as target samples).

$$\mathrm{Classification}\;\mathrm{Sensitivity}\;=\frac{\mathrm{TP}}{\left(\mathrm{FN}+\mathrm{TP}\right)}\;\times \;100\%$$

Specificity is the fraction of non-target samples correctly classified as non-target samples.

$$\mathrm{Classification}\;\mathrm{Specificity}\;=\;\frac{\mathrm{TN}}{\left(\mathrm{TN}\;+\mathrm{FP}\right)}\times \;100\%$$

Continuous variables are presented as the mean ± standard deviation. Student’s t-test and Duncan’s test were used for statistical analysis. A *p* value of less than 0.05 was considered to be statistically significant.

## 3. Results and Discussion

### 3.1. Removal of Outlier Samples

**Figure 1:**
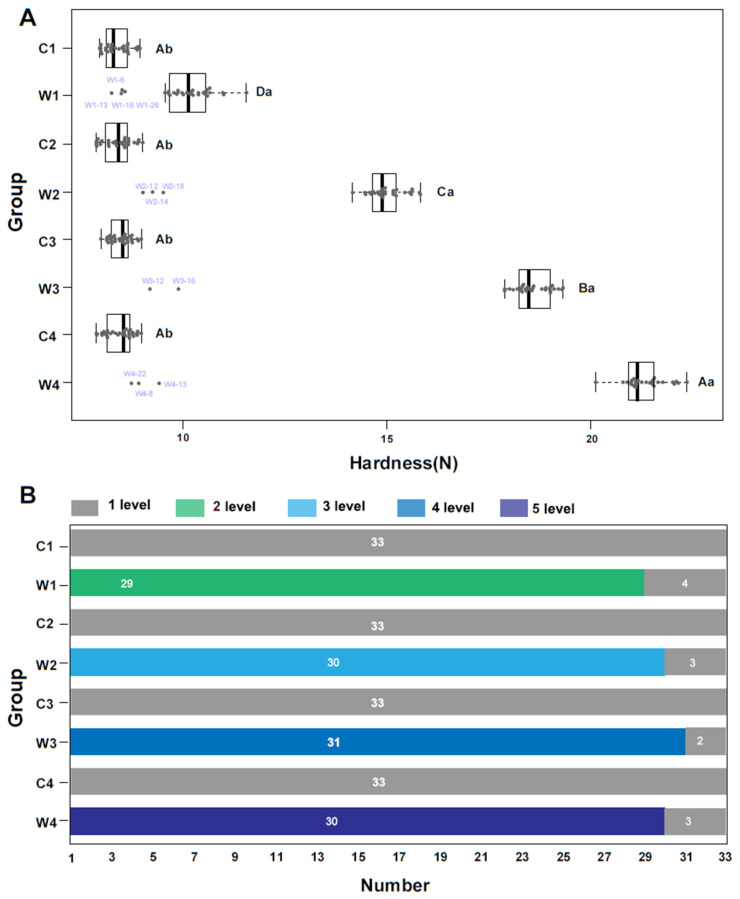
Removal of outliers using muscle hardness. C1, C2, C3, and C4 represent the ordinary grass carp from 30, 60, 90, and 120 day, respectively. W1, W2, W3, and W4 represent the crisp grass carp from 30, 60, 90, and 120 day, respectively. (**A**) Boxplot of muscle hardness of crisp grass carp and ordinary grass carp during different farming stages. Boxes represent the interquartile range, and whiskers delineate ± 1.5 times the interquartile range beyond the box boundaries. Points falling outside of the whiskers were considered outliers in this study. Statistical analyses were performed using Student’s *t*-test and Duncan’s test. Different lowercase letters represent significant differences in muscle hardness (*p* < 0.05). Different uppercase letters represent significant differences in muscle hardness (*p* < 0.05). (**B**) Sensory texture evaluation of crisp grass carp and ordinary grass carp during different farming stages. Samples from 33 fish were evaluated for each group.

Boxplots can be used to detect and eliminate outliers from a dataset [27]. This is an important step in machine learning-based analysis because outliers can misdirect the training process and produce a less accurate model [28]. As muscle hardness is the most obvious texture feature of crisp grass carp and increases with the faba bean feeding time [2], outliers were eliminated on the basis of muscle hardness. In general, there were a higher number of outliers in crisp grass carp than ordinary grass carp (Figure 1A). The boxplot revealed 12 crisp grass carp outliers (out of 132; 9.09%) exceeding the interquartile range by ± 1.5 times. The number of outliers varied across the feeding periods, where four outliers were found at 30 day (W1), three found at 60 and 120 day (W2 and W4, respectively), and two found at 90 day (W3).

The sensory evaluation results, which are known to be consistent with those of instrumental texture analyses in crisp grass carp evaluation [4], can be seen in Figure 1B. All the ordinary grass carp samples (132 fishes) were evaluated as level 1. For the crisp grass carp group, at 30 day, 29 samples were evaluated as level 2; at 60 day, 30 samples were evaluated as level 3; at 90 day, 31 samples were evaluated as level 4; at 120 day, 30 samples were evaluated as level 5; the remaining 12 samples were evaluated as level 1. These evaluation results were consistent with the result of the boxplot analysis (Figure 1A). Upon eliminating the outliers, 252 observations were retained for further analysis.

### 3.2. Blood Indexes Analysis

**Figure 2:**
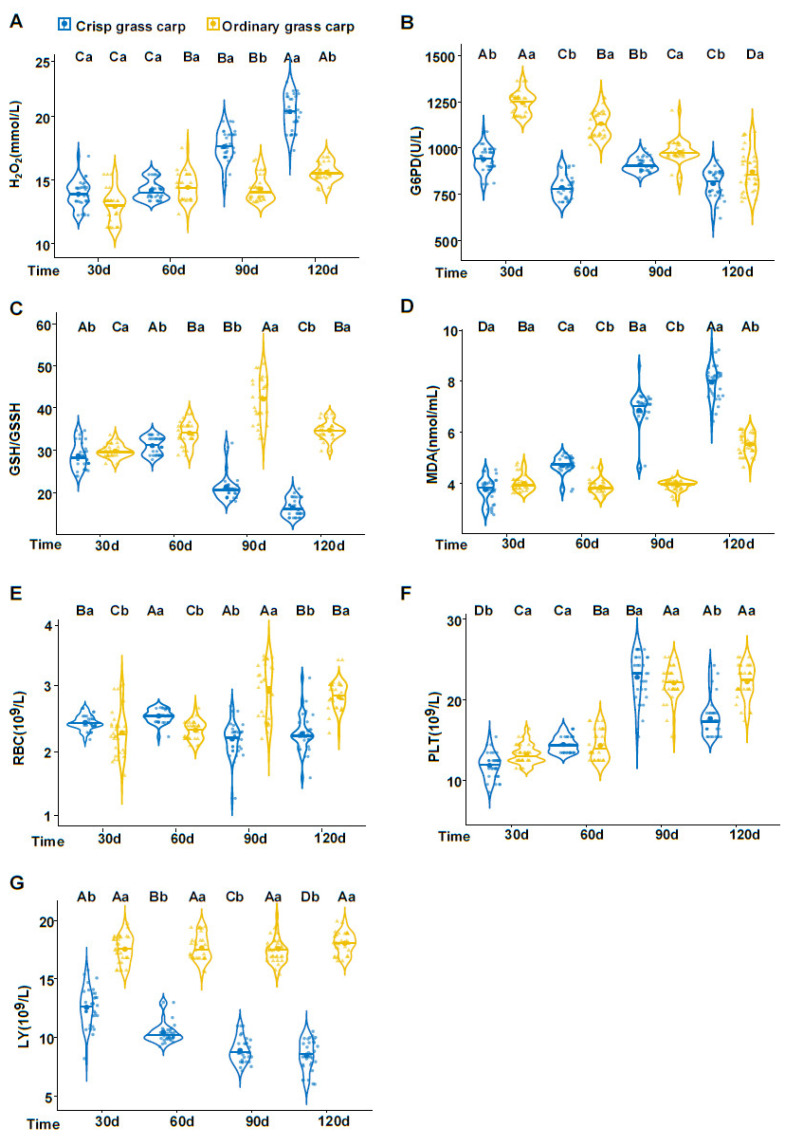
Violin plots of seven blood indexes in ordinary and crisp grass carp samples. The medians are represented by a horizontal blue or yellow line. The whiskers span the entire range. (**A**) H_2_O_2_. (**B**) G6PD. (**C**) GSH/GSSH. (**D**) MDA. (**E**) RBC. (**F**) PLT. (**G**) LY. Statistical analyses were performed using Student’s *t*-test and Duncan’s test. Different lowercase letters represent significant differences in blood indexes. Different uppercase letters represent significant differences in blood indexes (*p* < 0.05).

Our previous study found that faba bean suppresses the immune and antioxidant responses of grass carp [2,29]. To include the effects in the predictive models, seven blood indexes including blood red cells (BRC), platelet counts (PLT), lymphocyte (LY), hydrogen peroxide (H_2_O_2_), 6-phosphate dehydrogenase (G6PD), glutathione (GSH/GSSH), and malondialdehyde (MDA) were selected for this study. All values from 252 samples (120 crisp grass carp and 132 ordinary grass carp) are shown in the violin plots (Figure 2), in which some differences were observed depending on the culture periods and treatment. H_2_O_2_ levels were not significantly different between both groups on 30 and 60 day, but were higher in crisp grass carp than ordinary grass carp at 90 and 120 day (Figure 2A). The levels of G6PD, GSH/GSSH, and LY of crisp grass carp were significantly lower than those of ordinary grass carp throughout the culture period (Figure 2B,C,G). MDA markedly increased between 30 and 120 day in crisp grass carp and was notably higher than ordinary grass carp at 60, 90, and 120 day (Figure 2D). Compared to ordinary grass carp, the RBCs of crisp grass carp were significantly higher at 30 and 60 day but were lower at 90 and 120 day (Figure 2E). The PLT values between the two groups were also significantly different during the entire culturing period (Figure 2F). These differences were used to establish the classification models.

### 3.3. Natural Clustering Based on PCA Analysis

**Figure 3:**
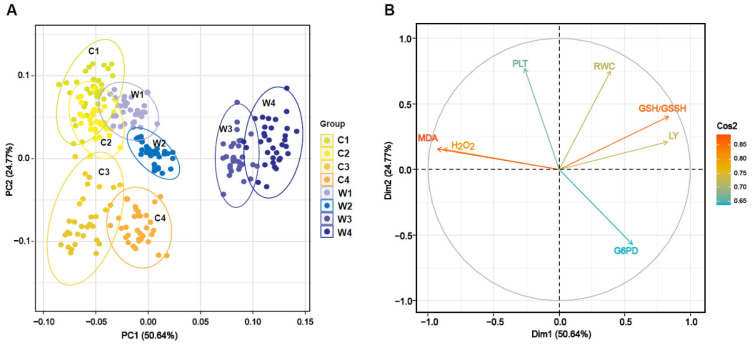
Natural clustering of crisp grass carp and ordinary grass carp. (**A**) Scatter plot of PCA scores from ordinary grass carp and crisp grass carp at different culturing stages. C1, C2, C3, and C4 represent the ordinary grass carp from 30, 60, 90, and 120 day, respectively. W1, W2, W3, and W4 represent the crisp grass carp from 30, 60, 90, and 120 day, respectively. (**B**) Loading plot for the original variables in the first two principal components (PCs).

The unsupervised PCA was used for the exploratory data analysis. The PCA model was built using the blood index data from 252 grass carp samples (120 crisp grass carp and 132 ordinary grass carp). Most of the variance in the data could be visualized in the first a few principal components (PCs). PC1 and PC2 represented 50.64% and 24.77% of the variance from the original data, respectively (Figure 3A). From the PCA plots, samples originating from W1, C1, and C2 strongly overlapped and showed negative scores in PC2. There was a slight separation between the centroids of the W1 and W2 samples. W3 samples slightly overlapped W4 samples and had positive scores in the PC1. There were marked differences between the centroids of W1, W2, W3, and W4 samples, especially between W2 and W3, which indicated that long-term feeding with faba bean exerted an obvious effect on blood indexes. In contrast, C3 and C4 samples slightly overlapped in the negative side of the PC2. The orientation of the variables in the PC2–PC1 plane is observed in Figure 3B. PC1 was strongly influenced by positive contributions from GSH/GSSH and LY and by negative contributions from MDA and H_2_O_2_. The dominant variables in PC2 included PLT and RBC.

The application of PCA allowed for a natural grouping of the 252 of grass carp samples, with a slight tendency of some samples to group more favorably. However, this approach could not systematically separate samples due to the overlap. Therefore, we subsequently applied several supervised methods classify crisp grass carp samples with different textures based on their blood indexes.

### 3.4. Classification and Comparing Classification Performance

Six machine learning techniques, GBDT, ANN, SVM, PLSR, NB, and RF, were applied to predict muscle hardness levels. The training and testing datasets were formed by 60% (152) and 40% (100) of the total samples, respectively.

**Figure 4:**
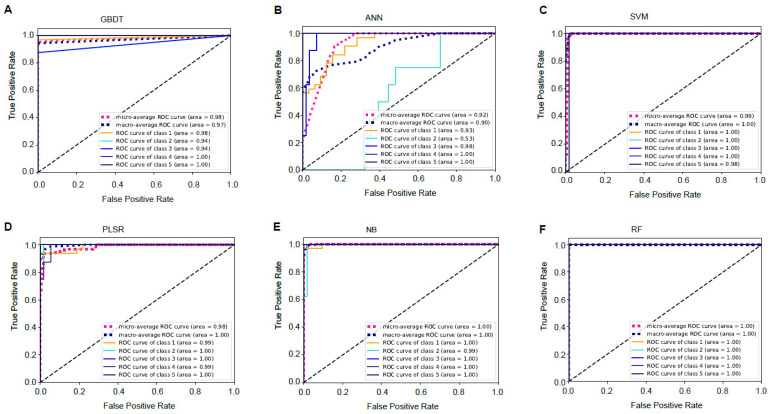
Receiver operating characteristic (ROC) curves for the six machine learning models. (**A**) Gradient boosting decision tree model. (**B**) Artificial neural network model. (**C**) Support vector machine model. (**D**) Partial least squares regression model. (**E**) Naïve Bayes model. (**F**) Random forest model.

In our analysis, the area under the receiver operating characteristic curve (ROC) was obtained for all models to aid in evaluations. The ROC represents a model’s success across varying discrimination thresholds, with the AUC representing the overall probability of correct classification. The shape of the ROC curve also provides insight into the model’s success [30]. The AUC of a binary class prediction could not be considered since our investigation involved a five-class prediction. Thus, micro- and macro-averages were used to obtain the ROC curves [31]. For the testing set, the AUCs of the micro- and macro-averages, respectively, were as follows: GBDT, 0.98 and 0.97 (Figure 4A); ANN, 0.92 and 0.90 (Figure 4B); SVM, 0.99 and 1 (Figure 4C); PLSR, 0.98 and 1 (Figure 4D); NB, 1 and 1 (Figure 4E); and RF, 1 and 1 (Figure 4F).

A confusion matrix (Table 1 and Table 2) was used to compare the discrimination performances of the six prediction models. The prediction accuracy of RF was the highest (96.01%) followed by NB (94.0%), PLSR (92.00%), GBDT (92.00%), SVM (91.00%), and ANN (89.00%). It is interesting to underline that the RF model exhibited the best prediction performance, with a classification accuracy of 100% for discriminating the crisp grass carp sampled across different culturing stages, while other models could not properly classify all of the target/authentic samples. The NB model incorrectly classified three observations from crisp grass carp with the third level hardness. GBDT and PLSR models correctly classified all but four crisp grass carp samples. ANN and SVM displayed lower classification performance with accuracies of 85.42% and 87.50%, respectively. RF demonstrated excellent average sensitivity (100%) and average specificity (93.08%) in classification of crisp grass carp samples.

In sum, the RF, NB, PLSR, and GBDT models all presented excellent accuracy (>90%), being capable of separating crisp grass carp samples into classes. The RF model was superior to other models as it allowed the discrimination with an accuracy of 100%. However, the present study has several limitations that need to be addressed. First, we only used data from our experimental facility, and the results should be validated using samples from other culture ponds or conditions. Additionally, increasing the number of samples will enhance the reliability of the machine learning models. Both of these limitations are currently being addressed in our research group.

## 4. Conclusions

This study established six machine learning-based approaches for the classification of muscle hardness in different crisp grass carp samples based on seven blood indexes (H_2_O_2_, G6PD, GSH/GSSH, MDA, RBC, PLT, and LY). The results showed that the RF model has the highest classification accuracy of 100%, followed by the NB model (93.75% accuracy), whereas the ANN model was the least accurate (85.42%). This approach provides an objective, cheap, fast, and reliable method that could help producers and consumers in evaluating the quality of in vivo crisp grass carp. Moreover, this system can be easily applied to evaluating the muscle quality of other freshwater fishes in vivo.

## Figures and Tables

**Table 1:** Confusion matrix of GBDT, ANN, and SVM classification for crisp grass carp. (**a**) GBDT. Accuracy of all samples = 92.00%; Accuracy of crisp grass carp samples = 91.67%. (**b**) ANN. Accuracy of all samples = 89.00%; Accuracy of crisp grass carp samples = 85.42%. (**c**) SVM. Accuracy of all samples = 91.00%; Accuracy of crisp grass carp samples = 87.50%.

(a) GBDT model								
Target	Level 1	Level 2	Level 3	Level 4	Level 5	Sensitivity	ASC	ASA
level 1	48	3	0	1	0	92.31%	-	91.80%
level 2	0	12	0	0	0	100%	92.00%	
level 3	3	1	8	0	0	66.67%		
level 4	0	0	0	12	0	100%		
level 5	0	0	0	0	12	100%		
Specificity	94.12%	75.00%	100%	90.91%	100%	-	-	-
APC	-	91.485				-	-	-
APA	92.01%					-	-	-
**(b) ANN model**								
**Target**	**Level 1**	**Level 2**	**Level 3**	**Level 4**	**Level 5**	**Sensitivity**	**ASC**	**ASA**
level 1	48	3	0	1	0	92.31%	-	86.79%
level 2	1	9	2	0	0	75.00%	85.00%	
level 3	0	2	10	0	0	83.33%		
level 4	0	0	0	10	2	83.33%		
level 5	0	0	0	0	12	100%		
Specificity	97.95%	64.26%	83.33%	90.91%	85.71%	-	-	-
APC	-	81.05%				-	-	-
APA	84.43%					-	-	-
**(c) SVM model**								
**Target**	**Level 1**	**Level 2**	**Level 3**	**Level 4**	**Level 5**	**Sensitivity**	**ASC**	**ASA**
level 1	49	2	0	1	0	94.23%	-	85.51%
level 2	0	8	3	0	0	66.67%	83.00%	
level 3	0	3	8	0	0	66.67%		
level 4	0	0		12	0	100%		
level 5	0	0	0	0	12	100%		
Specificity	100%	61.54%	72.73%	92.31%	100%	-	-	-
APC	-	81.65%				-	-	-
APA	85.32%					-	-	-

Note: ASC—average sensitivity of crisp grass carp samples; ASA—average sensitivity of all testing samples; APC—average specificity of crisp grass carp samples; APA—average specificity of all testing samples. Accuracy is the proportion of properly predicted samples to the total sample. Sensitivity and specificity are calculated as described in the Materials and Method. Green color denotes correct classification, red color denotes false classification.

**Table 2:** Confusion matrix of PLSR, NB, and RF classification for crisp grass carp. (**a**) PLSR. Accuracy of all samples = 92.00%; Accuracy of crisp grass carp samples = 91.67%. (**b**) NB. Accuracy of all samples = 94.00%; Accuracy of crisp grass carp samples = 93.75%. (**c**) RF. Accuracy of all samples = 96.00%; Accuracy of crisp grass carp samples = 100%.

(a) PLSR model								
Target	Level 1	Level 2	Level 3	Level 4	Level 5	Sensitivity	ASC	ASA
level 1	48	3	0	1	0	92.31%	-	91.79%
level 2	0	12		0	0	100%	92.00%	
level 3	3	0	9	0	0	75.00%		
level 4	0	0	0	11	1	91.66%		
level 5	0	0	0	0	12	100%		
Specificity	94.12%	80.00%	100%	91.67%	92.31%	-	-	-
APC	-	91.00%				-	-	-
APA	91.62%					-	-	-
**(b) NB model**								
**Target**	**Level 1**	**Level 2**	**Level 3**	**Level 4**	**Level 5**	**Sensitivity**	**ASC**	**ASA**
level 1	49	2	0	1	0	94.23%	-	93.85%
level 2	0	12	0	0	0	100%	94.00%	
level 3	1	2	9	0	0	75.00%		
level 4	0	0	0	12	0	100%		
level 5	0	0	0	0	12	100%		
Specificity	98.00%	75.00%	100%	92.31%	100%	-	-	-
APC	-	91.83%				-	-	-
APA	93.06%					-	-	-
**(c) RF model**								
**Target**	**Level 1**	**Level 2**	**Level 3**	**Level 4**	**Level 5**	**Sensitivity**	**ASC**	**ASA**
level 1	48	3	0	1	0	92.30%	-	98.46%
level 2	0	12	0	0	0	100%	100%	
level 3	0	0	12	0	0	100%		
level 4	0	0	0	12	0	100%		
level 5	0	0	0	0	12	100%		
Specificity	100%	80.00%	100%	92.31%	100%	-	-	-
APC	-	93.08%				-	-	-
APA	94.46%					-	-	-

Note: Refer to Table 1 for abbreviations.
